# Evolutionary Dynamics of N-Glycosylation Sites of Influenza Virus Hemagglutinin

**DOI:** 10.1371/currents.RRN1001

**Published:** 2009-08-18

**Authors:** Joshua L. Cherry, David J. Lipman, Anastasia Nikolskaya, Yuri I Wolf

**Affiliations:** ^*^NCBI/NLM/NIH, Bethesda, MD and ^†^NCBI/NLM/NIH/DHHS, Bethesda, MD

## Abstract

The hemagglutinin protein of influenza virus bears several sites of N-linked asparagine glycosylation. The number and location of these sites varies with strain and substrain. The human H3 hemagglutinin has gained several glycosylation sites on the antigenically important globular head since its introduction to humans, presumably due to selection. Although there is abundant evidence that glycosylation can affect antigenic and functional properties of the protein, direct evidence for selection is lacking. We have analyzed gain and loss of glycosylation sites on the side branches of a large phylogenetic tree of H3 HA1 sequences (branches off of the main, long-term line of descent). Side branches contrast with the main line of descent: losses of glycosylation sites are not uncommon, and they outnumber gains. Although other explanations are possible, this observation is consistent with weak selection for glycosylation sites or a more complicated pattern of selection. Furthermore, terminal and internal branches differ with respect to rates of gain and loss of glycosylation sites. This pattern would not be expected under selective neutrality, but is easily explained by weak selection or selection that changes with the immune state of the host population. Thus, it provides evidence that selection acts on the glycosylation state of hemagglutinin.

## Introduction

Influenza virus hemagglutinin is a trimeric glycoprotein that constitutes most of the surface protein of the virus particle.  The protein binds to sialic acid residues on host cells and mediates entry of the virus into the cell (reviewed in [1]).  Hemagglutinin is the most important protein for the host's protective antibody response.  Changes to the hemagglutinin sequence can therefore change the antigenic properties of the virus, allowing it to escape antibodies produced in response to earlier infections or vaccinations ("antigenic drift").  The human H3N2 hemagglutinin appears to be subject to strong selection for antigenic novelty, which leads to rapid evolution at many positions in the sequence.

Certain asparagine residues on the protein are modified by addition of oligosaccharide chains.  The number and location of these N-glycosylation sites varies with the strain and substrain.  The glycosylation state has been observed directly for only a few substrains.  However, likely glycosylation sites can be inferred from the protein sequence on the basis of a simple sequence pattern (N-X-[ST]-X, where X is not proline).

The glycosylation state of the protein may have a variety of selectively important consequences for the virus.  Oligosaccharides may shield the protein from the humoral immune response, providing an advantage even in naive hosts.  Changes in glycosylation state can be a source of antigenic novelty.  Addition of glycosylation sites to hemagglutinin can reduce or abolish binding by monoclonal antibodies [2] [3] or human antisera [4].  In one case [3], abolition of binding was shown to be due to glycosylation *per se* rather than the underlying amino acid change.  As discussed in greater detail below, loss of a glycosylation site might also create useful antigenic novelty.  Even if, as reported by [5], changes in glycosylation state are not associated with transitions between "antigenic clusters", it seems likely that they are often associated with significant antigenic change.  We note, in this regard, that the clusters analyzed by [6] span several units of antigenic distance, and many of them contain multiple vaccine strains, indicating that changes in the vaccine were deemed necessary due to within-cluster antigenic drift.  Additional effects of glycosylation on the immune response may also be selectively important: glycans can be targets of the innate immune system [7], and variation in the presence and nature of oligosaccharides might provide within-host diversity without sequence variation.  Glycosylation can also affect several non-immune-related aspects of hemagglutinin function, such as receptor affinity and the efficiency of the escape of new virus from host cells [4] [8] [9].  Thus, several different selective forces may act on glycosylation.  These forces may conflict, and the net fitness effect of gain or loss of a glycosylation site may depend on the ever-changing immune state of the host population.

The HA1 of the human H3N2 virus has experienced a long-term net gain of glycosylation sites since the appearance of H3 in humans in 1968.  This gain of sites, and their long-term maintenance, are presumed to be due to a selective advantage of glycosylation, although they might result from a fortuitous excess of gains over losses.  Here we examine, using a large phylogenetic tree of human H3 HA1 sequences, the dynamics of glycosylation sites along "side branches" of the tree (offshoots of the long-term line of descent).  The pattern along these branches contrasts markedly with the long-term pattern: losses of glycosylation sites are not uncommon, and they far outnumber gains.  This observation, though perhaps surprising, is not inconsistent with a long-term advantage of glycosylation.  In fact, the details of the pattern of gains and losses provide evidence that selection acts on the state of glycosylation.

## Results and Discussion

### Phylogenetic Tree

The analysis presented here is based on a large tree of human H3 HA1 nucleotide sequences and an associated ancestral state reconstruction.  This tree is an updated version of the one described in [10].  It was inferred using maximum parsimony, and the sequence reconstruction is a most parsimonious reconstruction.

The tree, as is usual for human H3, can be divided into a long trunk (a series of branches and nodes that corresponds to the long-term line of descent) and a series of offshoots that emmanate from it and lead to the terminal nodes.  For the portion of the tree corresponding to recent history, the trunk cannot be reliably identified; only time will tell which clade will be the source of future viruses.  The analysis presented here therefore uses a subset of the tree for which the trunk is identifiable.  This includes 174 trunk nodes.  Upper limits on the dates of these nodes range from 1968 for the root node to November of 2007 for node 174.  Terminal node dates range from 1968 to September 2008.

### Glycosylation Changes along the Trunk

Inferred sequence changes along the trunk of the tree confirm that, as has long been appreciated, there has been a net gain of glycosylation sites.  At the root we find six sites.  Through seven gains and two losses, the total becomes eleven.  One site, included in those tallies, is gained and subsequently lost along the trunk.  Of the original six sites, only three were in the antigenically important membrane-distal region of the protein molecule.  In contrast, all of the sites that were gained along the trunk, including the one subsequently lost, were in the distal region.  This pattern suggests that selection has favored gains of glycosylation sites on the membrane-distal region since the introduction of H3 to humans, most likely because of effects of glycosylation on antigenic properties.

### Gains and Losses on Side Branches

Along side branches, in marked contrast to the trunk, losses outnumber gains by nearly a factor of five (Table 1).  Losses of sites are not uncommon, and 24% of isolates in the tree have lost one or more glycosylation sites during their descent from the trunk.

**Table 1.** Numbers of gains and losses of glycosylation sites on trunk and side branches. 

**Side Branches vs. Trunk**

**Table:** 

	Trunk	Branches
Gains	7	51
Losses	2	248
	3.5:1	1:4.9

**** Why, if the pattern on the trunk indicates selection for glycosylation, is loss of glycosylation sites prevalent on side branches?  Perhaps the excess of gains over losses on the trunk is due to chance rather than selection.  However, the observed pattern is consistent with selection for glycosylation.  If loss of a glycosylation site is weakly deleterious, lost sites among isolates are expected to be more common than fixation of losses.  Because losses, according to this hypothesis, do not lead to severe fitness effects, they may be found among circulating viruses.  They are, however, most probably doomed to ultimate failure: small fitness effects are sufficient to make it extremely unlikely that such a virus becomes the ancestor of all future viruses.  Similar reasoning arises in interpretation of the McDonald-Kreitman test ([11]).  The converse argument holds for advantageous changes, and thus might apply to gains of glycosylation sites.   The observed pattern is also compatible with more complicated types of selection.  It might be, for example, that the loss of a glycosylation site initially confers an advantage.  As hosts are exposed to viruses that lack the site, and develop increased immunity to them, this advantage decreases, eventually becoming a disadvantage.  A short-term advantage might result from an improvement in hemagglutinin function that outweighs the initially small disadvantage of decreased protection from the humoral immune response.  Alternatively, the short-lived advantage of loss might come from the evasion of antibodies.  Although the protective effects of glycosylation are usually emphasized, it is conceivable that loss of a glycan would lead to antibody escape.  Despite the availability of only a few crystal structures, one antibody [12] is known to make contact with a hemagglutinin glycan, and several antibodies to influenza virus neuraminidase are known to make contact with a glycan on that protein [13] [14] [15].  For some of the anti-neuraminidase antibodies, evidence exists for a contribution of the glycan to the free energy of binding [16] [17].  Thus, loss of a glycosylation site might give rise to an antigenically novel protein that, stripped of its novelty, is inferior, and that therefore has a short-term advantage but a long-term disadvantage.   Another possibility that must be considered is that the excess of losses reflects selection for loss during growth of the virus in the laboratory.  It seems unlikely that such artifacts would plague one in four isolates (the fraction that have lost glycosylation sites), but this is a formal possibility.  Although [18] observed losses of the same glycosylation site during laboratory growth of two isolates, these observations were made after eight passages through cell culture, far more than are typically made before sequencing.  The site that was lost (at position 133) accounts for only five of the losses in our tree, and exclusion of this site from the analysis has negligible effect on the results.  Evidence that the excess of losses that we observe on side branches is not an artifact of selection in the laboratory is presented below.   

### Internal vs. Terminal Branches

Non-trunk branches may be divided into terminal branches and internal branches.  Terminal branches, unlike internal branches, lead directly to leaf nodes (actual sequences).  Sequence changes that occur during laboratory propagation are expected to be confined to terminal branches, so internal branches should be unaffected by any such changes.  Furthermore, assuming that such artifacts are rare, comparison of internal to terminal branches yields information about selection on glycosylation sites, as explained below.

The number of gains and losses on non-trunk internal and terminal branches are shown in Table 2. Losses exceed gains on both classes of branches.  However, the effect is much stronger in terminal branches than in internal branches.  The difference between internal and terminal branches is statistically significant according to Fisher's exact test (p=0.0003).

**Table 2.** Numbers of gains and losses of glycosylation sites on non-trunk internal and terminal branches.

**Internal vs. Terminal Branches**

**Table:** 

	Internal	Terminal
Gains	24	27
Losses	52	196
	1:2.2	1:7.3

****   The fact that the excess of losses over gains is seen on the internal branches is evidence that it is not an artifact of laboratory selection.  In an accurate reconstruction, sequence changes that occur in the laboratory would all appear on terminal branches.  In practice, parallel laboratory losses could be erroneously placed on internal branches by the reconstruction procedure.  However, this would require not only loss of the same glycosylation site by closely related sequences, but loss through the same nucleotide change.  Thus, such cases should be rare.The higher ratio of losses to gains on terminal branches, taken at face value, is evidence of selection.  If losses are mildly deleterious, they will be observed disproportionately on terminal branches (compared to neutral, advantageous, or less deleterious changes).  This is so because losses that can be observed will tend to have occurred relatively recently, since time tends to decrease their frequency.  The converse holds for advantageous changes, and might apply to gains.  Thus, selection against losses would lead to the observed pattern.  A more complicated pattern of selection, such as a short-term advantage for losses, could also produce this pattern. The difference in the ratio of losses to gains between internal and terminal branches might result from a difference in the rate of loss, a difference in the rate of gain, or some combination of these.  The total length of the terminal branches, as measured by synonymous nucleotide changes, is greater than the lenght of internal branches by a factor of 1.92.  It follows that the difference in ratio is due to roughly equal contributions of a difference in rates of loss and a difference in rates of gain: on terminal branches, as compared to internal branches, there is a 1.96-fold higher rate of loss and 1.71-fold lower rate of gain.  This observation supports selection against losses (at least in the long term) and selection in favor of gains.  

## Conclusion

Side branches of the tree differ qualitatively from the trunk with respect to glycosylation sites in that losses outnumber gains and are not infrequent.  This does not imply a lack of selection on glycosylation sites: if selection against loss is sufficiently weak, or if loss has a short-term advantage, this pattern would be expected.  The fact that losses are found disproportionately on terminal branches, and gains are found disproportionately on internal branches, is evidence that selection does operate.  In addition to providing information about the short-term trajectory of hemagglutinin sequences, these observations provide support for the view that the long-term gains and persistence of glycosylation sites reflects selection for glycosylation rather than chance.  A significant fraction (24%) of isolates have lost glycosylation sites, and attention to these losses may be important for choosing vaccine strains.

## Funding information

This research was supported by the Intramural Research Program of the NIH, NLM, NCBI.

## Competing interests

The authors have declared that no competing interests exist. 
